# Erythropoietin (EPO)-receptor signaling induces cell death of primary myeloma cells in vitro

**DOI:** 10.1186/s13045-016-0306-x

**Published:** 2016-08-31

**Authors:** Thea Kristin Våtsveen, Anne-Marit Sponaas, Erming Tian, Qing Zhang, Kristine Misund, Anders Sundan, Magne Børset, Anders Waage, Gaute Brede

**Affiliations:** 1 K.G. Jebsen Centre of Myeloma Research, Department of Cancer Research and Molecular Medicine, Faculty of Medicine, NTNU, Norwegian University of Science and Technology, Trondheim, Norway; 2 Present Address: Department of Cancer Immunology, Institute for Cancer Research, Oslo University Hospital, Oslo, Norway; 3 Present Address: Centre for Cancer Biomedicine, University of Oslo, Oslo, Norway; 4 Myeloma Institute for Research and Therapy, University of Arkansas for Medical Sciences, Little Rock, AR USA; 5 Centre of Molecular Inflammation Research, Department of Cancer Research and Molecular Medicine, Norwegian University of Science and Technology, N-7491 Trondheim, Norway; 6 Department of Immunology and Transfusion Medicine, St Olavs University Hospital, Trondheim, Norway; 7 Department of Haematology, St. Olavs University Hospital, Trondheim, Norway; 8 Department of Cancer Research and Molecular Medicine, Faculty of Medicine, Norwegian University of Science and Technology, NTNU, Trondheim, Norway

**Keywords:** Multiple myeloma, CD138^+^ cells, Erythropoietin, Erythropoietin-receptor, JAK-2, ERK-1/2, Bone marrow stroma cells, Co-culture, Survival

## Abstract

**Background:**

Multiple myeloma is an incurable complex disease characterized by clonal proliferation of malignant plasma cells in a hypoxic bone marrow environment. Hypoxia-dependent erythropoietin (EPO)-receptor (EPOR) signaling is central in various cancers, but the relevance of EPOR signaling in multiple myeloma cells has not yet been thoroughly investigated.

**Methods:**

Myeloma cell lines and malignant plasma cells isolated from bone marrow of myeloma patients were used in this study. Transcript levels were analysed by quantitative PCR and cell surface levels of EPOR in primary cells by flow cytometry. Knockdown of EPOR by short interfering RNA was used to show specific EPOR signaling in the myeloma cell line INA-6. Flow cytometry was used to assess viability in primary cells treated with EPO in the presence and absence of neutralizing anti-EPOR antibodies. Gene expression data for total therapy 2 (TT2), total therapy 3A (TT3A) trials and APEX 039 and 040 were retrieved from NIH GEO omnibus and EBI ArrayExpress.

**Results:**

We show that the EPOR is expressed in myeloma cell lines and in primary myeloma cells both at the mRNA and protein level. Exposure to recombinant human EPO (rhEPO) reduced viability of INA-6 myeloma cell line and of primary myeloma cells. This effect could be partially reversed by neutralizing antibodies against EPOR. In INA-6 cells and primary myeloma cells, janus kinase 2 (JAK-2) and extracellular signal regulated kinase 1 and 2 (ERK-1/2) were phosphorylated by rhEPO treatment. Knockdown of EPOR expression in INA-6 cells reduced rhEPO-induced phospo-JAK-2 and phospho-ERK-1/2. Co-cultures of primary myeloma cells with bone marrow-derived stroma cells did not protect the myeloma cells from rhEPO-induced cell death. In four different clinical trials, survival data linked to gene expression analysis indicated that high levels of EPOR mRNA were associated with better survival.

**Conclusions:**

Our results demonstrate for the first time active EPOR signaling in malignant plasma cells. EPO-mediated EPOR signaling reduced the viability of myeloma cell lines and of malignant primary plasma cells in vitro. Our results encourage further studies to investigate the importance of EPO/EPOR in multiple myeloma progression and treatment.

**Trial registration:**

[Trial registration number for Total Therapy (TT) 2: NCT00083551 and TT3: NCT00081939].

## Background

Multiple myeloma is a malignancy of plasma cells in the bone marrow. Interaction between the malignant plasma cells and stromal cells in the bone marrow microenvironment is assumed to support growth and survival of these cancer cells. This process is characterized by increased microvessel density induced by production of pro-angiogenic molecules and suppression of angiogenic inhibitors, a phenomenon called an ‘angiogenic switch’ [1]. Erythropoietin/erythropoietin-receptor (EPO/EPOR) signaling is the main regulator of proliferation in the erythroid lineage [2]. However, it recently became apparent that the EPOR also is expressed in several non-haematopoietic tissues including the central nervous system, retina, heart, vascular endothelium, kidney, lung, liver and gastrointestinal and reproductive tracts where it plays a role in the protection from apoptosis and inflammation induced by hypoxia, toxicity or injury [3–5]. The EPO/EPOR interaction initiates a signaling cascade that activates and recruits a variety of Src homology-2 (SH2) domain-containing proteins that initiate various downstream signaling pathways such as ERK-1/2 and JAK-2 [6]. These signaling pathways control cell proliferation, differentiation and/or death, dependent on the cell type and context of stimulation. The majority of myeloma patients are anaemic and administration of rhEPO to anaemic patients with advanced myeloma is associated with prolonged survival [7] and improved immunological functions [8]. Expression of EPOR mRNA in plasma cells from myeloma patients has been shown previously [9], and it was recently shown that primary myeloma cells expressed EPOR on the surface [10]. However, these studies did not address whether the EPOR was active in EPO signaling or whether it could affect primary myeloma growth or viability in vitro. The role of EPO/EPOR is still unclear in the pathogenesis of multiple myeloma. We show here that EPO/EPOR signaling is functional in both primary myeloma cells and cell lines and that recombinant human EPO exhibits a negative effect on myeloma cell viability in vitro.

## Results

### EPOR mRNA expression in myeloma cell lines and primary myeloma cells

**Fig. 1:**
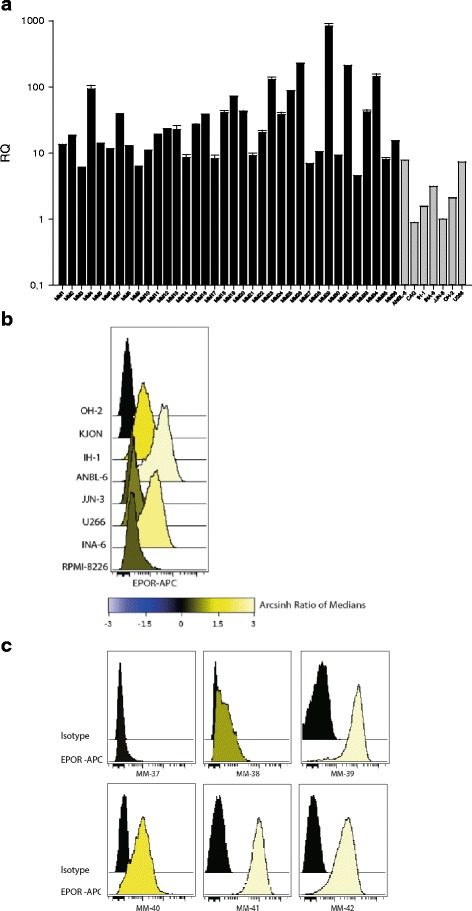
The EPOR is expressed both at mRNA and protein levels in primary myeloma cells and cell lines. **a** Quantitative real-time PCR for EPOR in 36 patient samples (*black*) and seven HMCLs (*gray*). Relative quantification (*RQ*) was calculated using the ΔΔCt method with GAPDH as housekeeping gene. CAG HMCL was set to 1. *Error bars* indicate standard deviation of triplicates for each sample. **b**, **c** Flow cytometry was used to detect surface EPOR levels in myeloma cell lines and in primary myeloma samples. The data are Arcsinh transformed showing the Archsinh value of medians, and negative OH-2 is used in the first row for comparison for the cell lines

The relative levels of EPOR mRNA in purified CD138^+^ cells from 36 myeloma patients and in seven human myeloma cell lines (HMCLs) were quantified with qPCR. Both the patient samples and the HMCLs showed EPOR expression although to variable extent (Fig. 1a). In order to investigate whether there was an autocrine EPO/EPOR signaling in myeloma cells, we measured expression of EPO mRNA in 20 of the samples. All samples tested were found negative for EPO mRNA (data not shown), suggesting that plasma cells do not normally express significant amounts of EPO and that EPO signaling in myeloma may occur through a paracrine or endocrine mechanism.

To examine whether EPO mRNA expression was a specific trait of malignant plasma cells, we used publicly available data sets to compare expression in plasma cells from healthy people and from patients with various stages of plasma cell neoplasms. We downloaded and analysed data from the IA7 release of the CoMMpass data (https://research.themmrf.org), containing expression data from 484 multiple myeloma patients, and we found that EPO was not expressed in any of the myeloma patients (fragments per kilobase of exon per million fragments mapped (FPKM) mean 0.02;(Min:0; Max:0.73)). Similar to what we had observed, EPOR was expressed in many of the patients samples, although the expression levels varied between patients (FPKM mean 5.73;(Min:0.42; Max74.7)). In addition, data from the Oncomine database revealed a 2-fold increase in expression of EPOR mRNA expression comparing normal plasma cells with monoclonal gammopathy of undetermined significance (MGUS) in one study [11], as well as 1.8-fold increase from normal plasma cells to smouldering myeloma in another study [12].

### Presence of EPOR on the cell surface of myeloma cell lines and primary myeloma cells

Cell surface expression of EPOR on six myeloma cell lines was estimated by flow cytometry. IH-1, INA-6 and ANBL-6 expressed the highest levels of EPOR (Fig. 1b), whereas OH-2 and KJON were negative for EPOR. In isolated primary myeloma cells, the majority (5/6) of samples tested expressed EPOR on their surface with expression ranging from ‘low’ (MM-38) through ‘intermediate’ (MM-40) to ‘high’ expression (MM-39, MM-41 and MM-42) (Fig. 1c).

### Recombinant human EPO reduces the viability of primary myeloma cells and is antagonized by anti-EPOR antibodies in vitro

**Fig. 2:**
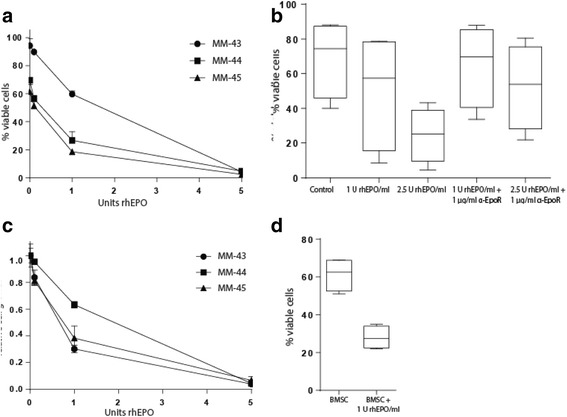
RhEPO-dependent reduction in cell proliferation and viability is counteracted by α-EPOR antibodies but not by stromal cells. Primary myeloma cells from three patients were treated with rhEPO as indicated and viability and proliferation were analysed. **a** Viability was measured by annexinV-FITC and PI staining with flow cytometry after 48 h treatment. *Error bars* represent variations of duplicates. **b** Proliferation was measured by cell ATP-release (CellTiterGlo assay) after 48 h treatment. *Error bars* represent variations within triplicates. Untreated sample is set to 1 for comparison. **c** Four primary myeloma cell samples (MM-44, MM-46, MM-47 and MM-48) were treated with 1 or 2.5 U/ml rhEpo in the presence or absence of neutralizing antibodies against EPOR as indicated. *Error bars* represents the variations of means of duplicates of the means of four patient samples. **d** Four primary myeloma samples (MM-44, MM-49, MM-50 and MM-51) were cultured with bone marrow stroma cells and 1 U/ml rhEPO. The bone marrow stroma cells did not protect the primary myeloma cells against the effect of 1 U/ml rhEPO. *Error bars* represents the variations of means of duplicates of means of four patient samples

To assess potential effects of EPOR signaling in myeloma cells, three primary myeloma cell samples were incubated with or without rhEPO for 48 h before cell viability and proliferation were measured using annexinV-FITC/PI and CellTiter Glo assays, respectively. Decreased viability and proliferation were observed with increasing concentrations of rhEPO (Fig. 2a, b), suggesting an inhibitory role of EPO on myeloma cells. In order to exclude the possibility of a rhEPO-independent cause for this observation, four additional primary myeloma cell samples were treated with rhEPO in the absence or presence of EPOR blocking antibodies. Antibody pretreatment to a large extent prevented rhEPO-induced cell death (Fig. 2c). This supports that the rhEPO is causing the cell death via the EPOR and not due to off-target effects.

Since bone marrow stroma cells has been shown to protect plasma cells against stress or pro-apoptotic agents in vitro, the effect of rhEPO on primary myeloma cells were analysed during co-culture with stromal cells. No protective effect in the presence of bone marrow-derived stromal cells was observed (Fig. 2d). Taken together, our results suggest that rhEPO reduces the viability of primary myeloma cells in a specific EPOR-dependent manner that cannot be prevented by stromal cells.

### EPO/EPOR signaling in myeloma cells

**Fig. 3:**
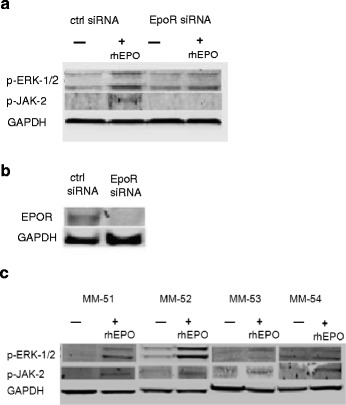
EPOR signaling in myeloma cells. **a** Knockdown of EPOR in INA-6 reduces p-ERK-1/2 and p-JAK-2 after rhEPO treatment (10 U/ml, 5 min). **b** Immunoblotting shows specific knockdown of EPOR in INA-6 cells using siRNA directed against EPOR mRNA. **c** Immunoblotting of four different primary myeloma cells after rhEPO treatment (10 U/ml, 5 min) shows an increase in both p-ERK1/2 and p-JAK-2 in the patient samples

To examine whether myeloma cells were able to signal via the EPOR, INA-6 cells were incubated with 10 U/ml rhEPO for 5 min and the levels of phosphorylated (p)-ERK-1/2 and p-JAK-2 were analysed by immunoblotting. Increases in phosphorylation of ERK-1/2 and JAK-2 were observed, suggesting that the EPO/EPOR signaling complex is functional in myeloma cells. Since ERK-1/2 and JAK-2 also can be activated by upstream signaling molecules other than the EPOR, the experiments were also performed after knockdown of the EPOR using siRNA. The levels of p-ERK-1/2 and p-JAK-2 after knockdown were reduced compared to the control-transfected cells exposed to rhEPO (Fig. 3a). The efficacy of the siRNA-mediated EPOR knockdown was examined by immunoblotting (Fig. 3b). We therefore conclude that INA-6 has the potential of functional and specific EPO/EPOR signaling. Examination of primary myeloma cells for their putative active EPO/EPOR signaling revealed that all samples (MM-51-MM-54) exhibited activation of both ERK-1/2 and JAK-2 after 5 min treatment with 10 U/ml rhEPO (Fig. 3c). Together, these results suggest that EPO/EPOR signaling may operate in myeloma cells.

### Higher levels of EPOR mRNA in malignant plasma cells are associated with prolonged patient survival

**Fig. 4:**
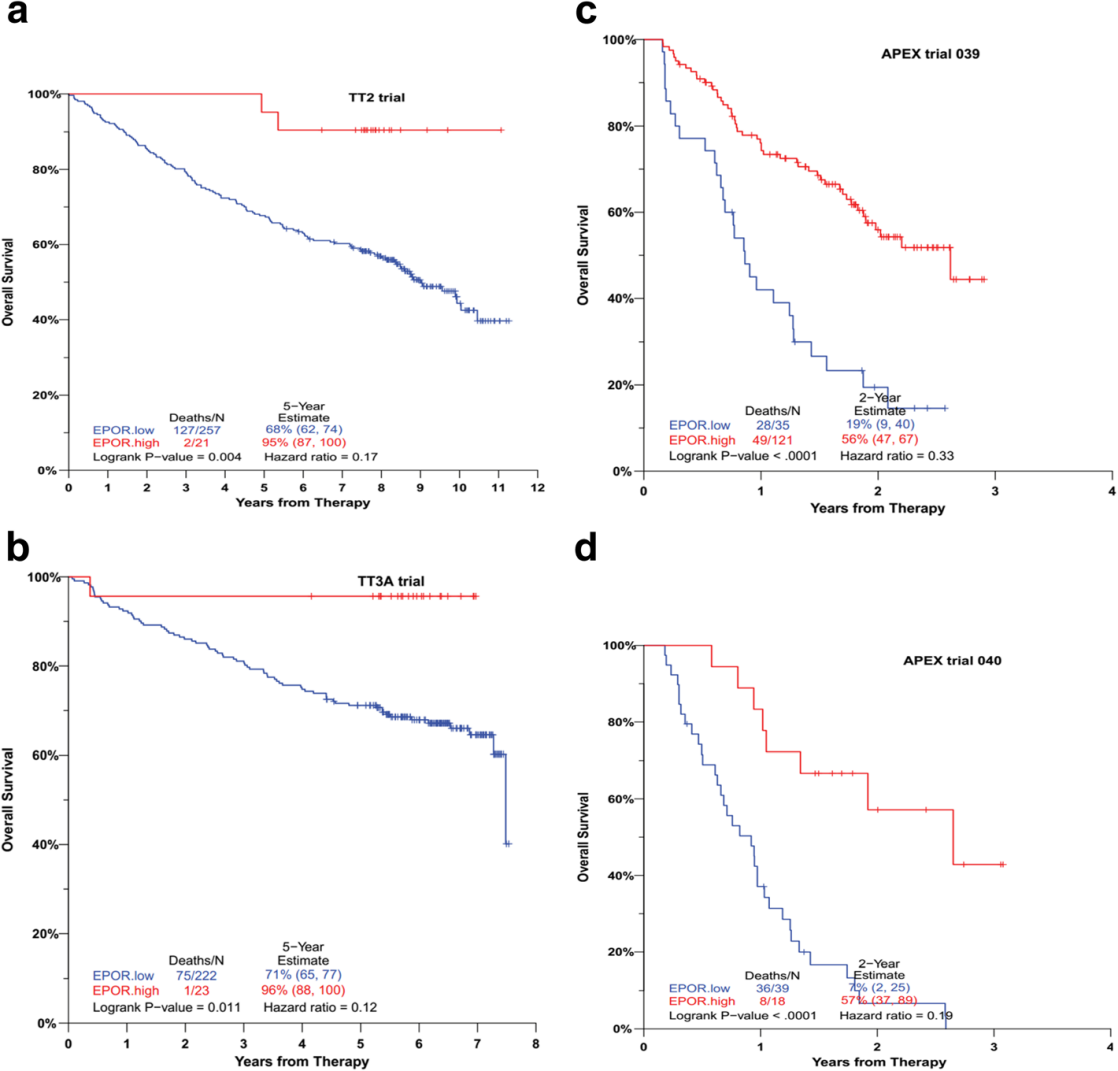
Prognostic relevance of EPOR expression from four independent patient cohorts. With the use of ROC optimal cutoff derived from each trial, overall survival analysis was performed on EPOR expression in the **a** TT2, **b** TT3A, **c** APEX trial 039 and **d** APEX trial 040 datasets. Low expression levels of EPOR adversely affect outcomes in all patient cohorts

We further asked whether plasma cell expression levels of EPOR was associated with clinical outcomes of myeloma patients. Kaplan-Meier analyses revealed significant differences in overall survival between ROC-high EPOR-expresser’s (*n* = 21) and the remainder of the patients (*N* = 257). High levels of EPOR favourably affected the outcome (Fig. 4). For newly diagnosed myeloma patients in the TT2 (*n* = 278) and TT3A (*n* = 245) trials, ROC-high EPOR expression levels were significantly associated with longer overall survival (*P* = 0.004, hazard ratio = 0.17; *P* = 0.011, hazard ratio = 0.12, respectively) (Fig. 4a, b). Similarly, for relapse myeloma patients with one to three prior therapies in the APEX phase 3 039 (*n* = 156) and 040 (*n* = 57) trials, higher expression levels of EPOR at relapse were significantly associated with better overall survival (*P* < 0. 0001, hazard ratio = 0. 33; *P* < 0. 0001, hazard ratio = 0.19, respectively) (Fig. 4c, d). The GEP data were collected at baseline prior to treatment.

## Discussion

Here, we demonstrate that the EPOR is expressed on the cell surface of myeloma cell lines and on primary myeloma cells, and show for the first time active EPO/EPOR signaling in malignant plasma cells. Activation of EPOR signaling had a negative impact on the viability of myeloma cell lines and of malignant primary plasma cells in vitro. The effect on viability was EPO/EPOR-specific since it could be partly reversed by neutralizing antibodies against EPOR. Treatment of myeloma cells with EPO was followed by JAK-2 and ERK-1/2 phosphorylation while knockdown of EPOR expression in the myeloma cell line INA-6 reduced JAK-2 and ERK-1/2 phosphorylation, again demonstrating the specificity of active EPO/EPOR signaling in myeloma cells. Co-cultures of primary myeloma cells with bone marrow-derived stroma cells did not protect the myeloma cells from EPO-induced death, while the stroma cells were unaffected. Together, our results suggest that EPO reduces the viability of primary myeloma cells in an EPOR-dependent manner and that the cells cannot be rescued by stroma cells. The level of EPOR mRNA in all primary samples as well as cell lines shows a variable level as measured by PCR. In the cell lines, like OH-2, EPOR was detected on mRNA levels but not on protein levels. This is most probably due to posttranscriptional regulatory mechanisms, causing EPOR expression levels below detection limits by flow cytometry.

In four different clinical trials, survival data linked to gene expression analysis indicated that higher level of EPOR mRNA was associated with better survival. Based on the survival studies, it is therefore intriguing to speculate whether active EPO/EPOR signaling may be favourable for the survival of myeloma patients. In agreement with this hypothesis, it is reported that therapy with rhEPO leads to longer overall survival in patients with multiple myeloma [13]. The use of EPOR-expression as a predictive biomarker to longer survival should be further explored. One could then get indications whether supportive treatment with rhEPO is safe with respect to bone density and whether it could be a potential to prolong survival in the patients with higher levels of EPOR expression. We believe, however, that it would be surprising if EPOR-expression can be a predictive biomarker on its own (reviewed in [14]).

A central concern, however, in the use of EPO to treat anaemia in cancer patients is whether tumour cells express EPOR and therefore could be stimulated to tumour growth. Also, concern has been raised whether endothelial cells that express EPOR could be activated by rhEPO exposure to induce angiogenesis and thereby indirectly promote tumour growth [15]. The use of rhEPO has therefore been tempered by adverse effects on cancer survival in recent clinical trials (reviewed in [16]).

It has been a concern that EPO, through its pro-proliferative effect in pre-erythrocytes, might enhance myeloma cell growth. Our data points in the opposite direction, especially for patients with high EPOR expression, favouring the use of EPO to treat anaemic patients with rhEPO. In another relevant study, EPO was used together with granulocyte colony stimulation factor (G-CSF) to see if eltrombopag (small non-peptide molecule that stimulates megakaryopoises in bone marrow) affected the proliferative capacity of human myeloma cells. These results showed no effect of EPO and G-CSF together in one cell line and some decrease of proliferation in another. They also show that drug combination did not reverse the apoptotic effect of lenalidomide or bortezomib, strengthening the fact that neither drug has pro-proliferative or anti-apoptotic effects [17].

In a newly published paper, lenalidomide was shown to increase EPOR levels by inhibiting the E3 ubiquitin ligase RNF41 in cells from the myeloid lineage involved in myelodysplastic syndrome. It is not known if this is also true for the plasma cells, but may open for a combinatorial treatment strategy between lenalidomide and EPO [18].

In another relevant study, it was found that mice (5T33MM murine myeloma model) treated with EPO showed accelerating bone resorption as measured by μ-CT, and was suggested to be caused by activation of bone marrow-derived macrophages [19]. These data were obtained in a mouse model and possible effects on bone density should be analysed in humans after EPO treatment. It would also be interesting to follow up and compare bone density in patients treated with both EPO and lenalidomide as a follow up from the Basiorka-studies [18]. A series of new upcoming treatments for myeloma is proposed and/or in clinical trials [20]. It would be interesting to analyse the effect of EPO as combinatory drug in at least some of these suggested treatment regimes.

Another intriguing observation from our study is the EPO-dependent activation of ERK, a typical hallmark of anti-apoptosis, which concurred with reduced viability. Most often, ERK and AKT pathways offer survival signals that protect cells from apoptosis [21]; however, increasing evidence reveals that ERK activation may contribute to apoptosis in certain cell types. For example, inhibition of ERK (by U0126) was renal protective in cisplatin-induced nephrotoxicity in mice [22], while in renal epithelial cells, cisplatin induces ERK activation and inhibition of ERK blocked apoptosis [23–25]. Furthermore, ERK activation contributes to apoptosis in OK cells following H_2_O_2_ treatment [26], while in a recent study, ERK activation was crucial in mediating triptolide-induced apoptosis [27]. Also in cultures of human neuroblastoma cells (h-NMB), EPO inhibits growth, while it stimulates differentiation [28]. Consequently, our observation that EPO-mediated ERK activation in myeloma cells is associated with reduced viability and adds to a growing list of instances of ERK activation concomitant with cell death. The JAK-2 activation and further trigging of apoptosis in the myeloma cells might become an issue if the myeloma patient is simultaneously treated with a JAK-inhibitor (e.g. AT9283 or ruxolitinib), and this should be investigated before treating patients in JAK-inhibitor clinical trials with supportive EPO therapy [29].

Our attempts to map possible EPO-dependent activation of p38 and JNK signaling pathways failed and mapping of these pathways is still inconclusive.

Together, this study shows that primary myeloma cells display active EPO/EPOR signaling and that rhEPO may induce myeloma cell death in vitro, both in monoculture and in co-culture of primary cells with myeloma-derived bone marrow stroma cells. Four independent survival studies establish a link between elevated EPOR-expression in myeloma cells and beneficial patient outcome and suggest that EPOR expression can be a novel prognostic marker for newly diagnosed myeloma.

## Conclusions

These results demonstrate for the first time that EPO/EPOR signaling is active in malignant plasma cells. Reduced viability and proliferation was observed both in cell lines and in primary myeloma cells upon treatment with rhEPO in vitro. The concomitant observation that higher EPOR expression is associated with survival in four different therapy strategies should encourage further studies to reveal the relevance of EPO/EPOR signaling in multiple myeloma progression.

## Methods

### Cell lines and primary multiple myeloma cells

The myeloma cell lines and CD138+ primary plasma cells from myeloma patients were grown and maintained as described [30]. Isolation of CD138+ cells for experiment demanding fresh viable cells and co-culture of CD138+ cells and bone marrow stromal cells were done as previously reported [30]. The project was approved by the Regional Ethics Committee (REK 2011/2029) and all patients has given informed consent.

### Transfection

For transient gene expression studies, INA-6 cells were transfected following the instructions for the Nucleofector device (Amaxa Biosystems, Cologne, Germany) using 2,5 μg CD4 plasmid, Hs_ EpoR_5 Flexitube siRNA, Hs_EpoR_6 siRNA (SI02780351, SI02780400, Qiagen, MD, USA) or control siRNA (Qiagen). The INA-6 cell line was used in the knockdown studies since it is transfectable and because it had a relatively high level of EPOR expression. After 24 h, transfected cells were isolated using dynaBeads anti-CD4 separation (Dynal, Oslo, Norway). Cells were treated with rhEPO (10 U/ml) (sigma E5627, 100,000 U/mg protein) 48 h after transfection.

### Real-time RT-PCR

RNA was isolated and cDNA was made as previously described [12] from primary patient samples and cell lines. TaqMan probes were used to detect the EPOR and EPO expression (Hs00959432_g1 and Hs01071096_g1, respectively, gene expression assays, Applied Biosystems, Foster City, CA). The comparative Ct-method was used for quantification with GAPDH (HS99999905_m1) as housekeeping gene on the StepOnePlus Real-Time PCR system (Applied Biosystems).

### Detection of EPOR on the surface of myeloma cells

EPOR on the surface of myeloma cells was stained with allophycocyanin (APC)-labelled anti-EPO receptor antibody [clone 12 K90] from Life Span Biosciences Inc (LS-C182845), 20 μl/ml for 30 min on ice after 20 min preincubation with human Fc receptor binding inhibitor (eBioscience, 50 μl/ml). Isotype control mouse IgG1 APC MG105 (Invitrogen) was used. All incubations were performed in PBS/1 % BSA. Flow cytometry was performed using LSR II (Becton Dickinson) with FACS Diva software (Becton Dickinson), and analysed with Cytobank [31]. Gates were set on live cells with forward and side scatter and duplets were excluded.

### Viability and proliferation assay

AnnexinV-FITC/PI staining (Abcam, ab14085) and analysis by flow cytometry was used to assess viability in the primary cells incubated with rhEPO in the presence or absence of anti-EPOR antibodies (Santa Cruz: sc101444, clone MM0031-6G7). The anti-EPOR was preincubated for 30 min together with the primary cells before rhEPO was added. Cell proliferation assay was performed after 48 h incubation with rhEPO using CellTiter-Glo® Luminescent Cell Viability Assay (Promega, Madison, Wisconsin) according to the manufacturer’s instructions.

### Western blot analysis

INA-6 was washed 4× in Hanks’ balanced salt solution, incubated for 4 h in 1 % BSA in RPMI before treatment with 10 U/ml rhEPO for 5 min. Primary samples were seeded in 1 % BSA in RPMI and starved for 4 h before treatment with rhEPO, 10 U/ml, 5 min. Lysates of INA-6 and primary cells were made as previously described [32]. Samples were subjected to electrophoreses and transferred to membranes by blotting. Membranes were then blocked with non-fat dried milk (5 %) in Tris-buffered saline supplemented with Tween-20 (TBS-T) (0.1 %) and incubated with antibody against p-ERK-1/2 (rabbit polyclonal IgG, Cell Signalling, 1:1000 and EPOR (sc101444, Santa Cruz, 1:200). For detecting p-JAK-2, membranes were blocked in 5 % BSA in TBS-T and incubated with antibody against p-JAK-2 (rabbit IgG anti p-JAK-2, Cell Signalling, 1:200). All antibodies were incubated up to 72 h at 4 °C. GAPDH (Abcam, Cambridge, UK, 1:50,000) was used as loading control. Antibody binding was visualized with Odyssey Licor detecting system using L680 anti-rabbit or L800 anti-mouse (1:20 000 in TBS-T).

### ScanR microscope-based screening and cell viability assay

Cell viability experiments of co-cultures were performed using a ScanR-automated fluorescence microscope (Olympus, Hamburg, Germany). Acquired images were analysed using the ScanR Image Analysis software. Image acquisition and analysis of myeloma cell viability was performed as described [30]].

### Gene expression profiling and statistical analysis

Gene expression data for newly diagnosed myeloma patients without prior therapy in the total therapy 2 (TT2) and total therapy 3A (TT3A) trials were retrieved from NIH GEO omnibus under accession number GSE2658 and EBI ArrayExpress under the accession number E-TABM-1138. Gene expression data for the relapsed myeloma patients with one to three prior therapies in the APEX phase 3 trial 039 and 040 trials were retrieved from NIH GEO omnibus under accession number GSE9782. There are six probe sets designated to EPOR (19p13.2) on both U133Plus2.0 and U133A microarray platforms. Among them, probe set 209962_at was consensually selected due to compatibility of two types of Affymetrix microarrays and oligo-sequence alignment to EPOR locus. Because the patients enrolled to TT2 and TT3A trials were treated under different regimens, the receiver-operating characteristic (ROC) optimal cut point for EPOR in each clinical trial was identified with the running log-rank test, respectively, based on overall survival of the cohorts at the time of data collection [33]. Hazard ratios were calculated by the Cox proportional model. All statistical computations were performed using R (version 2.13.2; available from http://www.r-project.org) and Bioconductor (available from http://www.bioconductor.org).

### Patients and sample collection of material for gene expression profiling in clinical trials

The information of Total Therapy (TT) 2 [NCT00083551 [34] and TT3 [NCT00081939 [35] trials were published at the web site of National Institutes of Health (https://clinicaltrials.gov). The baseline bone marrow aspirates were harvested from the patients at diagnosis of MM according to established procedures at the University of Arkansas for Medical Sciences (UAMS). Bone marrow specimens were processed to obtain CD138+ plasma cells for gene expression profiling [11]. The study was approved by the Institutional Review Board of UAMS, and informed consents were obtained from all study subjects and kept on record in accordance with the Declaration of Helsinki.

## Abbreviations

ERK1/2: Extracellular signal regulated kinase 1 and 2
FITC/PI: Fluorescein isothyocyanate/propidium iodide
JAK-2: Janus kinase 2 rhEPO: recombinant human erythropoietin
qRT-PCR: Quantitative reverse transcription polymer chain reaction
TT2/3: Total therapy 2/3

## References

[1] Raimondo F, Azzaro MP, Palumbo G, Bagnato S, Giustolisi G, Floridia P (2000). Angiogenic factors in multiple myeloma: higher levels in bone marrow than in peripheral blood. Haematologica.

[2] Krantz SB (1991). Erythropoietin. Blood.

[3] Lewis LD (2004). Preclinical and clinical studies: a preview of potential future applications of erythropoietic agents. Semin Hematol.

[4] Sasaki R (2003). Pleiotropic functions of erythropoietin. Intern Med.

[5] Buemi M, Cavallaro E, Floccari F, Sturiale A, Aloisi C, Trimarchi M (2003). The pleiotropic effects of erythropoietin in the central nervous system. J Neuropathol Exp Neurol.

[6] Chateauvieux S, Grigorakaki C, Morceau F, Dicato M, Diederich M (2011). Erythropoietin, erythropoiesis and beyond. Biochem Pharmacol.

[7] Mittelman M, Zeidman A, Kanter P, Katz O, Oster H, Rund D (2004). Erythropoietin has an anti-myeloma effect—a hypothesis based on a clinical observation supported by animal studies. Eur J Haematol.

[8] Prutchi-Sagiv S, Golishevsky N, Oster HS, Katz O, Cohen A, Naparstek E (2006). Erythropoietin treatment in advanced multiple myeloma is associated with improved immunological functions: could it be beneficial in early disease?. Br J Haematol.

[9] Kokhaei P, Abdalla AO, Hansson L, Mikaelsson E, Kubbies M, Haselbeck A (2007). Expression of erythropoietin receptor and in vitro functional effects of epoetins in B-cell malignancies. Clin Cancer Res.

[10] Miller CP, Rattray K, Zhang Y, Wood BL, Burwick N, Chien S (2012). Evaluating surface erythropoietin receptor in multiple myeloma. Leukemia.

[11] Zhan F, Hardin J, Kordsmeier B, Bumm K, Zheng M, Tian E (2002). Global gene expression profiling of multiple myeloma, monoclonal gammopathy of undetermined significance, and normal bone marrow plasma cells. Blood.

[12] Zhan F, Barlogie B, Arzoumanian V, Huang Y, Williams DR, Hollmig K (2007). Gene-expression signature of benign monoclonal gammopathy evident in multiple myeloma is linked to good prognosis. Blood.

[13] Baz R, Walker E, Choueiri TK, Abou Jawde R, Brand C, Mcgowan B (2007). Recombinant human erythropoietin is associated with increased overall survival in patients with multiple myeloma. Acta Haematol.

[14] Smith AD, Roda D, Yap TA (2014). Strategies for modern biomarker and drug development in oncology. J Hematol Oncol.

[15] Aapro M, Jelkmann W, Constantinescu SN, Leyland-Jones B (2012). Effects of erythropoietin receptors and erythropoiesis-stimulating agents on disease progression in cancer. Br J Cancer.

[16] Mckinney M, Arcasoy MO (2011). Erythropoietin for oncology supportive care. Exp Cell Res.

[17] Jeong J-Y, Levine MS, Abayasekara N (2015). The non-peptide thrombopoietin receptor agonist eltrombopag stimulates megakaryopoiesis in bone marrow cells from patients with relapsed multiple myeloma. J Hematol Oncol.

[18] Basiorka AA, Mcgraw KL, De Ceuninck L, Griner LN, Zhang L, Clark JA (2016). Lenalidomide stabilizes the erythropoietin receptor by inhibiting the E3 ubiquitin ligase RNF41. Cancer Res.

[19] Deshet-Unger N, Hiram-Bab S, Haim-Ohana Y, Mittelman M, Gabet Y, Neumann D (2016). Erythropoietin treatment in murine multiple myeloma: immune gain and bone loss. Sci Rep.

[20] Naymagon L, Abdul-Hay M (2016). Novel agents in the treatment of multiple myeloma: a review about the future. J Hematol Oncol.

[21] Yang JY, Michod D, Walicki J, Widmann C (2004). Surviving the kiss of death. Biochem Pharmacol.

[22] Jo SK, Cho WY, Sung SA, Kim HK, Won NH (2005). MEK inhibitor, U0126, attenuates cisplatin-induced renal injury by decreasing inflammation and apoptosis. Kidney Int.

[23] Arany I, Megyesi JK, Kaneto H, Price PM, Safirstein RL (2004). Cisplatin-induced cell death is EGFR/src/ERK signaling dependent in mouse proximal tubule cells. Am J Physiol Renal Physiol.

[24] Kim YK, Kim HJ, Kwon CH, Kim JH, Woo JS, Jung JS (2005). Role of ERK activation in cisplatin-induced apoptosis in OK renal epithelial cells. J Appl Toxicol.

[25] Nowak G (2002). Protein kinase C-alpha and ERK-1/2 mediate mitochondrial dysfunction, decreases in active Na + transport, and cisplatin-induced apoptosis in renal cells. J Biol Chem.

[26] Lee JS, Kim SY, Kwon CH, Kim YK (2006). EGFR-dependent ERK activation triggers hydrogen peroxide-induced apoptosis in OK renal epithelial cells. Arch Toxicol.

[27] Tan BJ, Chiu GN (2013). Role of oxidative stress, endoplasmic reticulum stress and ERK activation in triptolide-induced apoptosis. Int J Oncol.

[28] Degryse S, Cools J (2015). JAK kinase inhibitors for the treatment of acute lymphoblastic leukemia. J Hematol Oncol.

[29] Wollman Y, Westphal G, Blum M, Simantov R, Blumberg S, Peer G (1996). The effect of human recombinant erythropoietin on the growth of a human neuroblastoma cell line. Life Sci.

[30] Misund K, Baranowska KA, Holien T, Rampa C, Klein DC, Børset M (2013). A method for measurement of drug sensitivity of myeloma cells co-cultured with bone marrow stromal cells. J Biomol Screen.

[31] Kotecha N, Krutzik PO, Irish JM. Web-based analysis and publication of flow cytometry experiments. Curr Protoc Cytom. 2010 Jul; Chapter 10: Unit10.17. 10.1002/0471142956.cy1017s53 PMC4208272 20578106

[32] Holien T, Våtsveen TK, Hella H, Waage A, Sundan A (2012). Addiction to c-MYC in multiple myeloma. Blood.

[33] Florkowski CM (2008). Sensitivity, specificity, receiver-operating characteristic (ROC) curves and likelihood ratios: communicating the performance of diagnostic tests. Clin Biochem Rev.

[34] Barlogie B, Tricot G, Anaissie E, Shaughnessy J, Rasmussen E, van Rhee F (2006). Thalidomide and hematopoietic-cell transplantation for multiple myeloma. N Engl J Med.

[35] Usmani SZ, Sexton R, Hoering A, Heuck CJ, Nair B, Waheed S (2012). Second malignancies in total therapy 2 and 3 for newly diagnosed multiple myeloma: influence of thalidomide and lenalidomide during maintenance. Blood.

